# Approaches to implementing and financing primary health care in Kenya: a case of seven counties

**DOI:** 10.3389/frhs.2025.1298379

**Published:** 2025-02-07

**Authors:** Janette Karimi, Salim Hussien, Elizabeth Wangia, Mercy-Irene Kimani, Mohamud Mohamed, Melissa Wanda, Rosemarie Muganda, Rachel Ndirangu, Daniel Mwai, Mercy Wanjala, Fadhila Richter

**Affiliations:** ^1^ Division of Reproductive Maternal, Newborn Child and Adolescent Health (RMNCAH), Ministry of Health, Nairobi, Kenya; ^2^ Division of Primary Healthcare (PHC), Ministry of Health, Nairobi, Kenya; ^3^ Division of Health Financing, Ministry of Health, Nairobi, Kenya; ^4^ PATH Advocacy and Public Policy, PATH, Nairobi, Kenya; ^5^ Futures Health Economics and Metrics Ltd., Nairobi, Kenya; ^6^ Department of Human Anatomy, University of Nairobi, Nairobi, Kenya

**Keywords:** primary health care, primary health care networks, universal health coverage, health system strengthening, Kenya

## Abstract

**Background:**

Kenya has prioritized Primary Health Care as an indispensable foundation to realize UHC by 2022. Integral to this approach’s operationalization is the adoption of the primary health care networks (PCNs) model to strengthen service delivery efficiency and coordination. The PCNs are coordinated by a Multidisciplinary Team (MDT). The MDT is dynamic and should comprise a care and support team that matches patient health needs and the catchment population. This scholarly article delineates the outcomes of an investigative assessment reviewing the current state and trajectory of PHC implementation, focusing on the PCN implementation, and analyzing PHC financing modalities in 7 counties in Kenya.

**Methods:**

This study employed a mixed-methodological approach to gather data from seven counties; Garissa, Nyeri, Makueni, Vihiga, Kisumu, Nakuru, and Mombasa. Key informant interviews were conducted with county health officials and partners. Additionally, counties were supplied with templates for qualitative data. Data was subsequently analyzed using thematic analysis and descriptive statistics.

**Results:**

Successful implementation of PCNs was positively correlated with robust county-level leadership, prioritization of PHC funding, intersectoral collaboration, and joint planning initiatives. Counties which had achieved high levels of community health unit (CHU) establishment and functionality were more adept at successfully mapping and operationalizing PCNs. All participating counties adopted Sub-County Health Management Teams (SCHMTs) as the MDTs due to staffing limitations at primary care facilities consequently inhibiting the capacity for effective MDT engagement. Fiscal commitments at the county level were imperative for facilitating the mobility of MDTs and orchestrating community outreach initiatives. Reimbursements from the National Hospital Insurance Fund (NHIF) served as a pivotal financial conduit for the sustenance of primary care facilities.

**Conclusion:**

The study finds that robust leadership, funding, collaboration, and planning were crucial for the effective operationalization and financial structuring of PCNs. The study recommends that the county governments should invest more in PHC infrastructure, equipment, and supplies, as well as in strengthening the capacity and mobility of MDTs. The study also suggests that uptake of NHIF will enhance the sustainability of PCNs.

## 1 Introduction

Primary Health Care (PHC) serves as an indispensable cornerstone in the attainment of Universal Health Coverage (UHC), possessing the capacity to address more than 80% of population health requirements via an integrative approach encompassing preventive, promotive, curative, rehabilitative, and palliative care modalities. Moreover, PHC fosters equitable distribution of available resources across all levels, ensuring no economic burden is experienced by any citizen while accessing the health service delivery (1).

Following the Primary Health Care meeting in Astana, Kazakhstan, in 2018 (2), countries including Kenya have since renewed their commitment to Primary Health Care (PHC), with the government recognizing PHC as the best platform for the achievement of Universal Health Coverage (UHC). This has led to the realization of the paradigm shift from hospital and disease focus towards embracing preventive and promotive healthcare. In advancing its commitment to the realization of UHC, the ‘Ministry of Health (MOH) put in place enabling policies, including the Kenya Community Health Policy 2020–2030 (1), Kenya Primary Health Care Strategic Framework 2019–2024 (3), Community Health Strategy 2020- 2025 (4), and the Primary Health Care Network Guidelines (5) to guide mechanisms to strengthening PHC including community health systems.

Towards this end, the Ministry of Health has placed great focus on operationalizing primary health care services through establishing and rolling out primary health care networks (PCNs), the recommended approach for implementing the current Kenya Primary Health Care Strategic Framework 2019–2024. This framework outlines key strategic directions and new arrangements in formation of primary care networks that are in line with the WHO building blocks of health systems, i.e., leadership, management and governance, human resource for health, service delivery, health financing, commodity supply chain, infrastructure, health information, technology and innovation. A primary care network as defined in Kenya is a health service delivery unit comprised of a level 4 hospital as the hub and level 3(health center) and 2(dispensary) facilities as the spokes, which are in turn linked with community health units(CHU). This PCN is coordinated by a multidisciplinary team of healthcare workers selected from the hubs, spokes and CHU to jointly identify community health needs and respond to these needs through provision of preventive community health services and curative primary care services at the spokes, with streamlined referral to the primary referral level 4 hospital and beyond. The PCN model uses a person-centered approach to health and strengthens the linkage of health services from the community to primary healthcare facilities, making community members easily access the health services they require. Evidence shows that when countries adopt PCNs, efficiently coordinate the network of primary care practices, and strengthen communities of practice at the service delivery units; they can work at scale to provide a broad range of services and connect to higher levels of care (6). The Kenya PHC guidelines recommend at least one PCN per sub-county (3).

Despite evidence of the benefits of investing in PHC and commitments made by governments, its rollout in the transformation of health systems in Kenya has been slow (7). Little evidence indicates the counties successes and challenges in implementing these strategies. Therefore, this study aimed to assess the level of implementation of PCNs and other PHC-strengthening strategies in seven counties in Kenya. This document provides evidence of key aspects of PHC implementation based on the contextual realities experienced in the devolved units piloting this approach. The Ministry of Health envisaged that the documentation exercise would catalyze learning and, inform the scale-up of primary health care networks and strengthen PHC financing.

## 2 Methodology

### 2.1 Study design

The study involved a comprehensive mapping and documentation exercise to evaluate primary healthcare (PHC) implementation in selected counties within Kenya. The study design employed a mixed-methods approach, incorporating both qualitative and quantitative research methodologies.

The study involved a desk review of existing policy frameworks. Structured questionnaires were developed and administered through key informant interviews with key county health management team staff, and community health service providers. The structured questionnaires were designed, guided by the WHO's health system building blocks framework, the WHO's PHC measurement framework (8), the PHC Strategic Framework and The Primary Health Care Network Guidelines. Specific quantitative information about the different pillars of the health systems [i.e., Leadership and Governance, Health Financing, Service Delivery, Human Resources for Health (HRH), Health Products and Technologies (HPTs) and Health Information Systems] was collected through structured templates. Required quantitative information was derived from County specific health sector annual performance review reports for the financial year 2021/22. The information extracted was then sent to each of the 7 County Directors for Health and PHC focal persons for verification. A two-day validation meeting of the data collected was also held.

### 2.2 Sampling strategy and county selection criteria

The study employed purposive sampling to select the participating counties. Counties were selected based on their adherence to the established criteria. The criteria for county selection were based on various factors that reflect the readiness and capacity of each county to implement effective PHC strategies. The following criteria were used:

1. Existence of county specific policies and legislations: Counties were considered if they had adopted county specific policies and legislations to drive the implementation of primary healthcare. Examples of such policies include the Community Health Services Act and the Facility Improvement Fund Act.
2. Innovative and Sustainable Financing Models: Counties were selected if they demonstrated the presence of innovative and sustainable financing models that support PHC implementation.
3. Tailored Service Delivery Models: Counties that had adopted service delivery models tailored to the specific needs of their communities were included in the assessment.

### 2.3 Study location and target participants

The documentation exercise was conducted across seven counties in Kenya: Garissa, Nyeri, Makueni, Vihiga, Kisumu, Nakuru, and Mombasa. The chosen counties comprised sites from previous Universal Health Coverage (UHC) pilot counties (Nyeri and Kisumu) and PHC-PCN model non-pilot counties (Mombasa, Garissa, Vihiga, Makueni, and Nakuru).

The target participants for the PHC documentation exercise included key stakeholders at the county level. These stakeholders encompassed:

i. County Chief Officer
ii. County Director of Health (CDH)
iii. PHC focal person
iv. Community health personnel
v. Community Health Assistants (CHAs)
vi. County pharmacist
vii. County health records and information officer

### 2.4 Data collection team

Data collection for this assessment was carried out by a team of officials from the Ministry of Health Kenya, representatives from PATH, and external consultants. They had a clear understanding of the assessment's design, objectives, and ethical considerations, including obtaining county entry permissions and informed consent from participants. The data collectors were also well-versed in the interview guide questions and effective interviewing techniques.

### 2.5 Tools development and methods

Comprehensive data collection tools were developed and reviewed collaboratively by teams from the Department of Primary Health Care, PATH staff, and external consultants to facilitate the data collection process. Feedback and revisions were incorporated into the final versions of the tools before the commencement of data collection.

All interviews were conducted in English and took place at mutually convenient locations for the respondent and the interviewer. The average duration of the interviews ranged from 30 to 45 min. The majority of the interviews were conducted in person. Photographs were taken throughout the interview, and audio recordings were archived. The audio recordings and hard-copy questionnaires were securely stored, with access limited to the study team through password protection and physical key access.

### 2.6 Data analysis

Following data collection, quantitative data from structured questionnaires were subjected to descriptive statistical analysis. This involved summarizing the data using frequencies, percentages, and averages to provide a quantitative overview of the PHC implementation status. The gathered information was analyzed within the context of the identified health systems themes. Open-ended responses were coded and organized into themes that captured key findings, challenges, and emergent patterns related to PHC implementation. The collective data from the seven counties were compiled into a comprehensive report

## 3 Results

**Figure 1:**
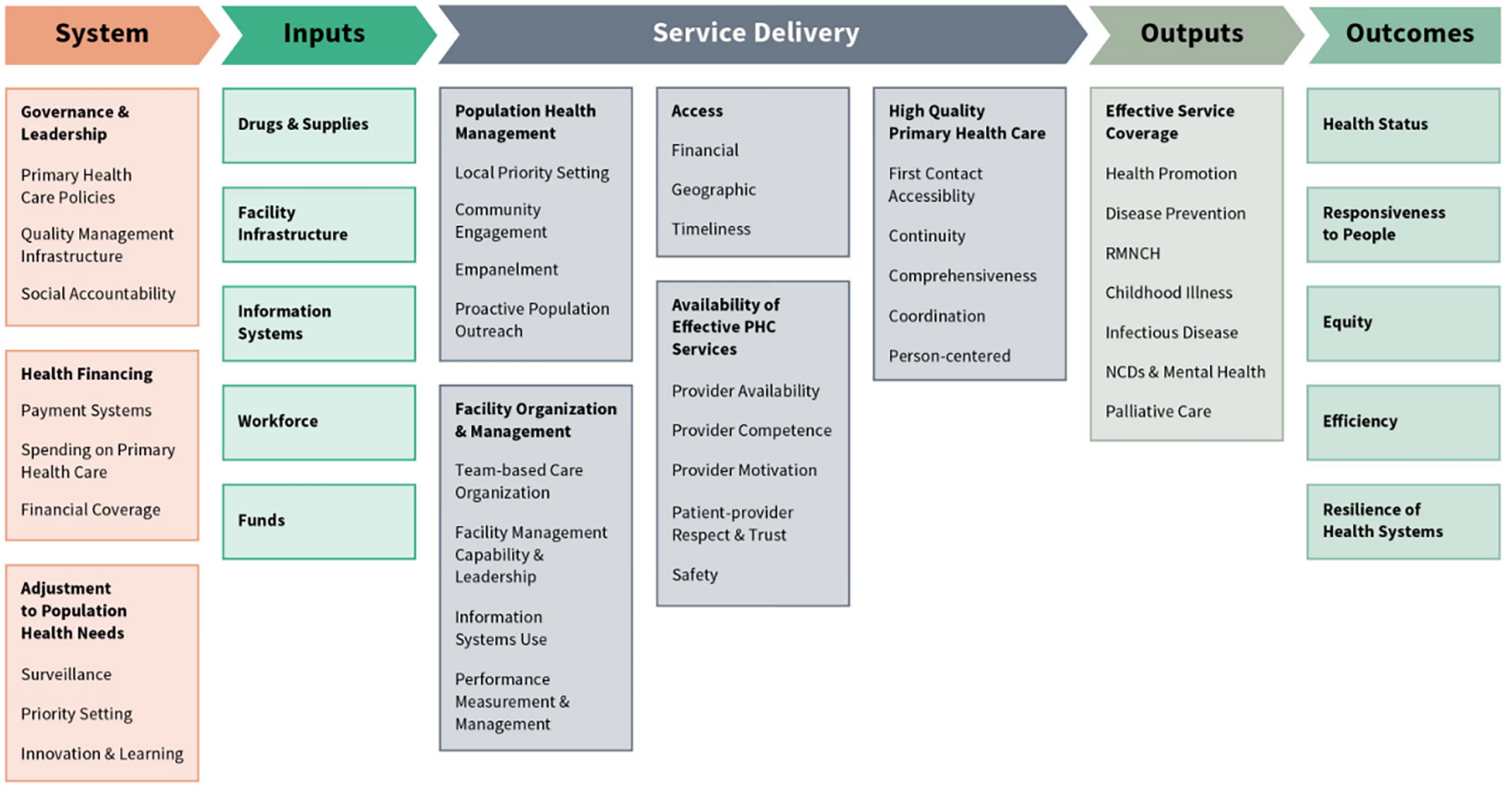
Primary healthcare performance initiative (PHCPI) conceptual framework (8).

The results follow the methodology framework derived from the WHO health systems building blocks and the Primary HealthCare performance initiative (PHCPI) conceptual framework (Figure 1). The inputs and the organization of inputs under leadership and resultant outcomes in terms of service delivery were assessed.

The overall PCN status in the Counties as of March 2023: Kisumu had established 4 functional PCNs, Nakuru had 4 functional PCNs, Garissa had 7 functional PCNs, Mombasa had 2 PCNs and had operationalized 3 satellite health centers of the Coast General Hospital, Makueni had 2 functional PCNs. Nyeri and Vihiga counties had no functional PCNs.

### 3.1 Governance

Implementing PCNs was associated with strong county leadership, as in the example of Kisumu and Nakuru, where, without partner support, the CHMT leadership was committed to success. Garissa was the first county to implement a PCN in all sub-counties due to strong leadership coupled with partner support. Makueni and Mombasa established PCNs in 2023 with support from the World Bank to conduct the baseline needs assessment and have subsequently funded the PCNs through the county budget. Vihiga had not yet established governance structures due to a lack of buy-in of the leaders and high leadership turnover. Nyeri had also not established PCNs.

Sensitization and training of all CHMT/SCHMT facilities and communities was an important success factor in setting up PCNS. This was the case in Kisumu, Nakuru and Garissa, where PCNs have been implemented. In Makueni, only the CHMT and SCHMTs had been sensitized on PCNs. In Vihiga, where only the CHMT had been sensitized on PCNs, the PCNs were yet to be operationalized. Nyeri CHMT had not been sensitized on PCNs; hence, none had been formed.

An appointed PHC focal person/office was key to ensuring the planning and implementation of PCNs. By the time of this assessment, all 7 counties had an appointed PHC focal person. In Mombasa, the PHC focal person oversaw the primary facilities coordination to conduct outreach services; however, PCN implementation had been driven by the sub-counties where the 2 PCNs were operational. Vihiga and Nyeri, despite having a PHC focal person, did not have PCNs due to other challenges outlined.

Embracing joint planning and distribution of resources within PHC led to more efficient use of resources, better coordination, and improved health outcomes. In Kisumu, a department of PHC was established, which housed the Community Health, Nutrition, Family Health, RMNCH, Mental Health, Cervical Cancer, and Child Health. In Garissa, the Family Health Directorate housed the Divisions of PHC, Reproductive Health, Nutrition, Child Health, Immunization, M&E, and quality assurance. This led to better coordination of PHC services.

Intersectoral collaboration and public participation in the health sector led to greater utilization of PHC services. All the counties had several partners supporting PHC interventions, which could also be leveraged to support PCN implementation and financing. The governance mechanisms are summarized in Table 1.

**Table 1:** PHC leadership and governance performance indicators per county.

	Kisumu	Nakuru	Garissa	Makueni	Nyeri	Vihiga	Mombasa
2. PHC Department/unit	Yes	Yes	Yes	Yes	Yes	Yes	Yes
3. PHC TWG established?	Yes	Yes	Yes,	Yes	No	No	No
4. Number of Functional PCNs (with MDT team, and implementing a work plan	4/8 (50%)	4/11 (36%)	7/7 (100%)	2/6 (33%)	0/8 (0%)	0/5 (0%)	2/6 (33%)
5. Nature of MDT	SCHMT serves as MDT	SCHMT serves as the MDT	SCHMT serves as the MDT	SCHMT serves as the MDT	N/A	N/A	SCHMT serves as MDT

#### 3.1.1 Challenges

The understanding of primary health care and health care networks differed across counties. Some County leaders understood PHC to be a program alongside others like immunization and reproductive health rather than an approach to health service delivery. Others understood PHC as being limited to community health services. This posed a challenge in broadly strengthening primary health care services at the community and health facility level. Those who understood the PHC strategy as per the Kenya Primary HealthCare Strategic Framework have been the counties that have implemented PCNs.

### 3.2 Human resources for health

The Kenya Primary Health Care Strategic Framework recommends forming multi-disciplinary teams. The MDT is envisioned to identify the disease burden and population health needs within their primary health care zone within which CHUs lie and respond appropriately. The MDT should also work with the CHVs linked to the network to ensure the appropriate referral of clients from the community unit to the linked PHC health facility.

For Kisumu, Nakuru, Garissa, Makueni, and Mombasa, the SCHMT doubled up as the MDT, led by the Sub county Medical Officer for Health (SCMOH) and incorporated specialists as needed. In Garissa, the sub-county nursing officers doubled up as the sub-county PHC coordinators. This was necessitated by the need to be cost-effective by using already existing staff. However, it was reported as a disadvantage since SCHMT members had competing managerial tasks, which negatively affected service delivery optimization.

The role of Family Health Clinicians was critical in the implementation of PHC. In Makueni, a county PHC focal person was a Family Medicine Specialist overseeing PCN implementation. Similarly, Kisumu had a family physician who previously led the piloting of the PCN model but was redeployed to model an NCD clinic at another location. Nonetheless, 7 Family Health-trained clinical officers supported and led the mini-MDTs. Nakuru had 4 Family Physicians, 1 in management, 2 in clinical practice at level 4 facilities and 1 in clinical training. It also had clinical officers trained in family medicine. Garissa had one family physician who oversaw PCNs. In Vihiga, the PHC focal person was a Family Medicine Specialist.

#### 3.2.1 Challenges

All the 7 counties had suboptimal numbers of HCWs, especially at primary-level facilities. This was a reported barrier to the formation of PCNs in Garissa and necessitated CHMT/SCHMT taking up administrative roles and doing clinical roles in Nakuru and Kisumu, Garissa, Makueni and Mombasa. The staff shortages were attributed to the high county wage bill, which had reached or surpassed the recommended proportion of the county total health expenditure (9) and the political interference of HRH recruitment. Most of the level 2 and level 3 facilities had only one staff, and their absence for one reason or another resulted in the closure of the facilities and disruption of essential services. The allocation of roles for family physicians in administration and only stationing them at one health facility was a barrier to having them set up and manage MDTs in Nakuru and Vihiga counties.

### 3.3 Service delivery

Strong CHUs are a foundation for strong community health systems and PCN implementation as seen in Kisumu with 88% CHU coverage, Garissa (78%), Nakuru (84%), Mombasa (82%) and Makueni (80%). The functionality of CHUs was reported based on monthly reporting by CHVs, and action days and dialogue days were carried out.

Despite Vihiga (97%) and Nyeri (100%) having high CHU coverage and strong community systems, the PCNs had not been formed due to other challenges. The findings on characteristics of CHUs are summarized in Table 2.

**Table 2:** Community health systems per county.

Indicator	Kisumu	Nakuru	Garissa	Makueni	Nyeri	Vihiga	Mombasa
Total formed Community Units	280 (88%)	362 (84%)	250 (78%)	240 (80%)	251 (100%)	146 (97%)	216 (82%)
Total Functional Community Units	280 (100%)	306 (85%)	210 (84%)	240 (100%)	251 (100%)	146 (100%)	194 (90%)
Total Community Health Committees	168 (60%)	55 (15%)	204 (82%)	0 (0%)	251 (100%)	88 (60%)	21 (10%)
Number of CHVs in the county	2,998 (94%)	3,175 (88%)	2,500 (78%)	3,722 (62%)	2,386 (95%)	1,446 (89%)	2,387 (90%)
Density of community health volunteers (per 5,000 population)	12 (120%)	6.6 (66%)	7.3 (73%)	17 (170%)	10 (100%)	12 (120%)	9 (90%)
Number of CHAs in the county	119 (37%)	28 (8%)	137 (55%)	20 (33%)	87 (35%)^a^	116 (77%)	25 (12%)

^a^ Nyeri County has 2 trained CHAs and 85 PHOs acting as CHAs.

#### 3.3.1 Challenges

The role of Community Health Committees (CHCs) in providing oversight to the CHU was not clear, with the functionality of CHCs being minimal or non-existent in the 7 counties. Nyeri had the highest number of CHCs, with all 251 CHUs having CHCs to oversee its management. Vihiga and Kisumu had CHCs established in 60% of the CHUs but with varying degrees of functionality. In Nakuru, 15% of the target CHCs were functional. Makueni and Garissa had no CHCs, while only 10% were active in Mombasa. A baseline assessment done in 3 Kisumu sub-counties revealed that the lack of a stipend for the CHCs led to the members stepping down to serve as CHVs to get a stipend. The roles of CHC members and CHVs were thought to be duplicative and not necessary for the functionality of the CHUs.

Although CHUs were linked to primary care facilities, no county had empaneled community members to a health facility. There were no mechanisms to prevent bypassing health facilities or ensure the community members went to their link facility.

The PHC facilities were not optimally equipped to meet the community health service needs, with shortages of commodities and staff leading to bypassing level 2–3 health facilities. This was the case in Kisumu, Garissa and Nakuru, which needed to equip some facilities to serve as hubs and spokes. In sub-counties with no level 4 facilities, some health centers were upgraded with infrastructure, equipment, and staff to serve as hubs.

### 3.4 Health financing

All 7 counties allocated more than 25% of the county budget to health. Mombasa had 27%, Garissa 28%, Vihiga 30%, Kisumu 33%, Nyeri 34%, Makueni 37%, Kisumu and, Nakuru 40%. The government contributed the most to the total health expenditure for all counties. All the indicators in Table 3 had the amount spent on a particular line item or program area divided by the total county health expenditure to generate the proportion of county health expenditure spent on the specific area. The bulk of county health expenditure went into paying salaries. Salaries ranged from 54% to 83% of total county health expenditure across the 7 counties. Different counties had different methods of accounting for the HRH salaries, with most categorizing it under administrative costs. Nakuru was the exception as it categorized HRH salaries based on where the staff worked (preventive, curative or administrative function). The spending of preventive promotive health services was low with Garissa allocating 8% of the county health expenditure, the highest among the 7 counties (Table 3).

**Table 3:** Health financing PHC performance indicators per county.

Budget item	Kisumu	Nakuru	Garissa	Makueni	Nyeri	Vihiga	Mombasa
% of County Health Expenditure (CHE) spent on development projects	1%	12%	12%	19%	5.1%	17%	19.3%
% of CHE spent on recurrent expenditure	99%	88%	88%	81%	94.9%	83%	80.7%
% of CHE spent on curative and rehabilitative services	10%	67% (including HRH) 29% (excluding HRH)	10%	19%	8.4%	18%	3% (The rest of curative budget is funded from FIF collected)
% CHE spent on preventive promotive services	1%	27% (including HRH) 7% (excluding HRH)	8%	7.5%	4.5%	4%	5.8%
% of CHE spent on General Administration, Planning, Management Support and Coordination * Amount spent on General Administration/CHE	89%	10% (including HRH) 2% (excluding HRH)	70%	73.5%	87.1%	78%	91.2%
% of CHE for HRH salaries (Amount spent on salaries/CHE)	83% Included in General Admin	56% Spread across the 3 program areas	60% Included in General Admin	59% Included in General Admin	81% Included in General Admin	54% Included in General Admin	67.8% Included in General Admin
% of CHE for HPTs Amount spent on HPTs/CHE	3.%	12% Included in preventive and curative services	5%	8.8%	8% Included in preventive and curative services	9%	2.4%

All the facilities from levels 2 to 5 were contracted by the National Health Insurance Fund (NHIF) and could claim from NHIF for both the regular cover and Linda Mama through the e-claims system (Table 4). In Kisumu, all the facilities were NHIF accredited, and a CHMT member was charged with oversight of NHIF claims. In Vihiga, all the facilities were NHIF accredited, and the disbursement of NHIF and Linda Mama funds to the facilities was timely with little or no delays. However, in Nakuru, despite the majority of the level 2 and 3 facilities being NHIF accredited, NHIF reimbursements were delayed. The primary care facilities in Garissa County had recently signed contracts with NHIF. In Mombasa, all government facilities except for 2 were NHIF accredited. In Nyeri, all Level 3s and 60% of Level 2s had been contracted by NHIF in the last 1 year. However, Nyeri had the lowest proportion of health facilities submitting NHIF claims (4%), followed by Garissa (20%) and Vihiga (49%). Most of the claims submitted by Nyeri and Vihiga were reimbursed. Counties like Mombasa, Makueni and Kisumu, which invested in equipment and personnel to process NHIF claims, had a higher proportion of facilities, including Level 2 and 3, submitting NHIF claims for Linda Mama and the national schemes. Kisumu had the largest proportion of claims reimbursed by NHIF. The status of enactment of facility improvement fund (FIF) County Bills to retain revenue collected at health facilities was as shown in the Table 5.

**Table 4:** NHIF funding per county.

Budget item	Kisumu	Nakuru	Garissa	Makueni	Nyeri	Vihiga	Mombasa
Proportion of all Government facilities claiming for Linda Mama	60%	58%	20%	77%	4%	49%	98%
Proportion of all Government facilities claiming for NHIF UHC/County indigent scheme	32%	No Data	10%	68%	4%	52%	98%
Proportion of all Government facilities claiming NHIF Super cover scheme	13.7%	68%	5%	74%	4%	40%	98%
% of NHIF claims reimbursed	80%	No Data	20%	63%	77%	75%	50%

**Table 5:** Health financing FIF bill and community bill per county.

County	1. FIF bill status	Specifics of FIF
Kisumu	Kisumu County Finance Bill 202, enacted	• Level 4 and 5 facilities to spend funds at source as per AWP and Annual Budgets by retaining 70% of FIF and sending 30% to the county revenue account. • The county allocates a budget to all health facilities from level 2 to level 5 health facilities
Nakuru	Nakuru County Public Finance Management (Hospital Management Services) Regulation, 2014, enacted	• The county FIF Act 2014 allowed the facilities to spend 100% of revenue generated at the source. • For (levels 4 and 5) 10% is sent to the County Health Revenue Fund for the facilitation of PHC services. • All revenue raised through Public Health Services, goes to CRF
Garissa	FIF draft bills awaiting ascent	• Level 4 and 5 facilities retain 100% of user fees. • Level 2 and 3 facilities use NHIF reimbursements in the provision of routine services
Makueni	The Makueni Finance Act 2020, enacted	• Allows Level 4 facilities to also retain 85% of revenue generated through user fees, and 15% is sent to the CRF to manage community health services including paying the CHVs Ksh 2000 stipends each, community dialogue days, action days, maternity open days, social marketing of the Makueni care program to promote its uptake and use among the community.
Nyeri	Nyeri Health Services Fund Act and Regulations 2021, enacted	• Level 4 & 5 facilities retain 80% at source and send 20% to the county for primary health care support.
Vihiga	Vihiga FIF Act 2019	• As per the Vihiga FIF Act, the level 4 hospitals were authorised to retain 70% of FIF, spend 3% on administration, 2% on emergencies while 25% was sent to the CRF to fund the CHMT/SCHMT activities
Mombasa	Amendment to the Mombasa Health Act 2017 in the county assembly on appropriation AIE for facilities to retain 100% FIF and not to remit to the CRF	• 100% revenue retention at the health facilities though it is not yet legislated

#### 3.4.1 Challenges

Generally, there was a low allocation of the County Health budget to primary health care in all counties. Budget allocation to preventive and promotive measures ranged from 1% in Kisumu to 8% in Garissa, the highest allocation among the 7 counties (Table 3). However, different counties had different budgeting methodologies, with counties like Nakuru distributing HRH and HPTs across the 3 program areas (curative, preventive and general administration. Most of the other counties classified HRH costs under general administration. Counties like Mombasa had a low proportion of county budgets on curative services because this was mostly catered for by user fees raised.

Lack of prioritization and funding for PCN-specific activities was a major hindrance to implementing PCNs in Makueni and Vihiga, where PCN mapping has been done but activation of PCNs was not yet in place. Nyeri County had not yet mapped PCNs and had no funding. Primary healthcare activities such as community health outreaches in Garissa County were highly dependent on partners' support.

Nakuru, Garissa, Mombasa, and Nyeri had experienced challenges in claiming NHIF and Linda mama reimbursements, especially at level 2 and 3 facilities, due to various reasons, including not having the required biometrics machines to institute claims and facilities not being aware of the claims processes. However, Makueni County received training on making NHIF claims and had timely reimbursements at all facility levels.

### 3.5 Health products and technologies

Kisumu, Nyeri, and Mombasa counties have made great strides in accelerating their efforts to ensure the availability of basic equipment, diagnostics, and essential medicines in level 3 and 2 facilities. The availability of essential medicines estimated for providing healthcare services was: Kisumu (47%), Mombasa (43%), Garissa (38%), Nakuru (44%), Vihiga (44%), Nyeri (53%) and Makueni (48%).

Autonomy of facilities to order directly from Kenya Medical Supplies Agency (KEMSA) improved commodity availability. This was linked to counties creating FIF bills to enable facilities to retain the revenue generated at the facility level to finance operational costs, including commodities and supplies.

#### 3.5.1 Challenges

All counties had experienced commodity stockouts, especially in the level 2 and 3 health facilities. Lack of commodities was cited as the major cause of bypassing primary care facilities to level 4 facilities. There was a limited supply of commodities for the Level 2 and 3 facilities due to the essential drug list that excluded NCD-related medication. Feedback from the client satisfaction surveys carried out in Kisumu at all levels revealed a lack of hypertensive and diabetic medication at level 2 and 3 facilities, which led clients to go to Level 4 facilities where they purchased the drugs out-of-pocket or via NHIF. This was also the case in Nakuru County.

The Level 2 and 3 facilities also faced challenges due to a lack of qualified personnel for commodity quantification and management. County debts with KEMSA also contributed to commodity budgets going into paying debts, thus having less to purchase commodities. The Level 2 and 3 facilities also faced challenges due to the lack of qualified personnel for commodity quantification and management.

### 3.6 Monitoring and evaluation

As a measure of supporting sustainable digitized community health systems, Kisumu County government and Living Goods partnered to develop an electronic community health information system (eCHIS) to mitigate the issues of inconsistent and low-quality reporting at the community level. CHVs in Kisumu are using eCHIS during household visits. Nakuru County was piloting an EMR system in 6 health facilities with plans for interoperability with KHIS and scaling up to the whole county. Most counties are conducting PHC M&E as part of the routine monitoring and evaluation systems in the county and therefore, this can be done under already existing structures. Nyeri trained CHV to use a COVID-19 household vaccination scorecard to monitor the uptake of the vaccine, and, similarly, an NCD household scorecard to monitor a number of hypertensive and diabetic people. Makueni adopted the Afya Kijijini project, where they used a digital tool to transmit hypertension and diabetes test results and prompt for a referral.

#### 3.6.1 Challenges

Despite PHC and PCN-specific indicators being outlined in the National M&E Plan and the PCN guidelines, counties were not yet monitoring using these indicators. The counties perceived the WHO and PHCPI frameworks as too detailed, difficult, and geared toward RMNCH. This was likely due to a lack of in-depth training on PHC monitoring and the need to understand that PHC is within the health system and not a parallel program.

There was a widespread shortage of community health reporting tools, with some counties overcoming this by photocopying the tools. Poor referral was due to a lack of MOH 100 community referral tool. None of the counties had implemented electronic medical record systems. The lower-level facilities lacked ICT equipment to facilitate data entry and monthly reporting. The lack of ICT equipment also limited the ability of primary health facilities to process NHIF electronic claims.

## 4 Discussion

The PHC model has been shown to be central to achieving SDG3, Universal health coverage, offering a direct link to health services to patients in need regardless of income status (10). Different counties have adopted different approaches and strategies to operationalize and finance PHC based on their contexts, needs, and resources (11). Primary healthcare networks PCNs) have emerged as one of the promising models for strengthening primary healthcare. Several studies in LMICs have shown evidence for primary healthcare networks in increasing access to care and improving the quality of healthcare and health outcomes and justify future studies (12). Networks of Care (NOCs) can improve quality of care, continuity of care, and maternal and newborn outcomes. They focus on relational elements that are key to health system functioning and are context-specific (13). This has been shown in studies in Indonesia and Cameroon, where MNH outcomes improved through the use of PCNs. The Indonesia NOC improved the capacity of providers and facilities in the network and clinical outcomes. In Cameroon, healthcare workers across several facilities formed a WhatsApp group to coordinate newborn referrals, forming the Perinatal Networks of Yaoundé. The approach helped improve access to care for neonates, formed trusting relationships among providers, which are key to its success, and it has incited a change in professional culture (13).

Strong community health units (CHUs) are a foundation for strong PCNs. CHUs are responsible for providing community health services, such as health promotion, disease prevention, and referral to primary care facilities (14, 15). There still, however, remain some challenges in community health systems, such as the lack of clarity and functionality of CHCs, the lack of recognition and remuneration of CHVs as an HRH cadre, the lack of empanelment of community members to a health facility, and the lack of referral mechanisms between CHUs and primary care facilities (14). Motivation of CHVs through mentorship and remuneration has been shown to strengthen the quality of PHC at the CHU level, as shown by a study in Uganda (16).

The same challenges faced in Kenyan counties have been seen in other parts of the world. These include low financing for PHCs, inefficient leadership, coordination and communication, shortage of HCWs, reluctance of providers to work together across different facilities and cadres and lack of commodities (17, 18). Therefore, investment in PHC and PCNs must take a health system strengthening approach.

Government commitment and coordination for PHC is critical to successfully implementing PHC systems. PHC has been demonstrated to work in other countries like Singapore (19), Cuba (20), Brazil (21) and Ghana (22), where there is national and subnational level commitment to coordination of primary health care networks. In Kenya, the success is also seen similarly by county-level leadership commitment. Counties which had a clear understanding of PCNs as envisioned and outlined in the PHC Strategic Framework and that train and sensitize their CHMTs, SCHMTs, facility managers, and community health stakeholders on PCN formation and management, implement PCNs successfully (23, 18) but to accelerate and strengthen primary care systems, there is a need for greater national-level commitment, guidelines and funding support to counties in PHC scale-up and implementation.

Financing for PHC: A robust health financing system centered on PHC is essential to the success of UHC provision (18, 21). Where PHC has been successful in other countries, robust health financing, including increased allocation of resources to running PHC facilities and expansion of insurance, has been implemented (10). Moreover, financial incentives to health workers have been shown to improve the quality of PHC health care (21). Funding for PHC in Kenya has been decreasing in the past decade, with the proportion of PHC expenditure from the Total Health budget dropping from 63.4% in the 2016/17 financial year to 53.9% in 2020/21. Moreover, external financing for PHC and healthcare has been decreasing over time from 28.3% to 23.9% over the same time period (18). As seen in the 7 study counties, funding directly to preventive promotive health services is often less than 5% of the county's total health expenditure. This drives a high reliance on household out-of-pocket expenditure as a means of funding PHC, with catastrophic effects on the households' financial well-being (24). For PCNs to succeed, the domestic financing towards PHC needs to gradually increase as Kenya continues to grow as a middle-income country. By shifting public facility financing away from reliance on user contributions and donor funding, county governments can improve health facility financing in Kenya (9).

The national government allocation to PHC facilities can be increased directly to cater for the reduced donor funding. There needs to be an increase in health insurance uptake in Kenya from 26% (KDHS 2022) and an expanded benefits package that caters for the full spectrum of care, from prevention to curative care, in order to protect the citizens from impoverishment as they seek care. This need has been supported by the success of PHC implementation in China, where ambitious health reforms were rolled out in 2009 to cater for its increasing population. Insurance coverage has since been increased to 95% of the populace. The likelihood of seeing a doctor increased from 16.2% in 2010 to 22.7% in 2016. Moreover, catastrophic health expenditure in low-income households dropped from 22.9% to 16.8% in the same time frame (25). Remarkable successes have also been noted in Denmark, Norway and Finland, where 85% of healthcare expenditure is catered for by taxation. Patient-doctor interaction has been reported to be as high as 85% (26).

The role of digitization in strengthening PHC is critical. Most countries have prioritized the digitization of higher-level facilities. Digitization provides an efficient platform to enable the community members be empaneled and mapped to a specific primary healthcare facility to receive services (27). Also, this will enable the enactment of a gatekeeping mechanism to ensure clients seek primary care services at lower-level facilities where it is cheaper, leaving secondary and tertiary hospitals to offer specialized services. The gatekeeping will also help facilities plan better for their catchment population and be better able to quantify and forecast commodity needs (3, 18, 28). Digital health systems are increasingly becoming important in improving patient referral and continuity of care.

Family health clinicians are critical in implementing PHC. Kenya has invested in training family physicians aimed to support PCNs. However, they have faced several challenges in delivering their mandates, including being posted to administrative roles among other challenges (29). Using already existing governance and HRH mechanisms to implement PCNs is useful, especially where resource constraints exist, as shown by its effectiveness in China (25). However, the challenge of suboptimal numbers and distribution of primary health workers, especially at primary-level facilities, often hinders the formation and functioning of PCNs (28); therefore, investment in adequate numbers and a mix of skilled healthcare workers is critical in attaining UHC. There is a need to change the mindsets of health workers and managers to embrace a team-based approach to delivering care and ensure that family physicians are optimally utilized in setting up and managing MDTs (28, 29). These are key factors for improving the quality and continuity of care and enhancing the satisfaction and motivation of health workers.

Appropriate technologies are among the pillars of PHC systems. Most countries have focused on equipping secondary and tertiary-level health facilities to the detriment of PHC facilities. This has resulted in patients often bypassing the PHC facilities to seek services in the higher-level facilities (30). The autonomy and capability of facilities to place direct orders with Kenya Medical Supplies Agency (KEMSA), which is designed to provide high-quality, reasonably priced medical supplies and equipment to all public health facilities, must be improved (15, 26, 27).

## 5 Limitations

The documentation exercise had some limitations that should be acknowledged and addressed in future studies. The exercise was conducted in only seven counties out of the 47 counties in Kenya, which may limit the generalizability and representativeness of the findings. The selection of the counties was also based on specified criteria that may introduce some bias or confounding factors, such as the existence of policies, legislations, financing models, or service delivery models that may not be applicable or available in other counties. Therefore, the results should be interpreted with caution when contextualized to similar settings. The research also relied mainly on qualitative data collection methods, such as key informant interviews, which the respondents' subjective opinions, perceptions, or experiences may influence. The questionnaires may also have overlooked some aspects or dimensions of PHC implementation that are not captured by the specific framework.

## 6 Recommendations

### 6.1 Governance and leadership

National and subnational (County) health management teams training on PHC is critical for a common understanding of PHC as a holistic, bottom-up approach to health and not as a subset of other programs or as a vertical program running alongside others. Subnational engagement through cross-county learning forums, especially on PHC innovations and building the capacity of counties to form structured communities of practice within the PCNs, will likely accelerate the scale-up of PCNs. Counties should priorities support for robust support supervision and on-the-job training, which are critical to institutionalize PCNs in the sub-counties.

### 6.2 Health financing

Counties should encourage facility financial autonomy by passing FIF bills to ensure the ring-fencing of revenue generated from health facilities and use these funds both for curative and primary health services. Additionally, counties should generally increase budgetary allocation to PHC services and set aside a budget for PCN-specific activities.

NHIF reimbursements are a key potential revenue source for health facilities. Counties need to invest in increasing population uptake of NHIF insurance to reduce out-of-pocket spending for households. Investment in infrastructure facilitating e-claims will significantly increase the turn-around time on claims processing. In addition, onboarding of clerks to ensure timely claims processing and training facility in charges on NHIF claims and financial management is key to maximizing reimbursements.

### 6.3 Service delivery

Each county should map out PCNs and implement them incrementally to all sub-counties based on lessons learned to enable the strengthening of community-facility linkages. This includes equipping community units to be fully functional and equipping level 2 and 3 health facilities to provide services per the KEPH norms and standards. The MDT should be composed of SCHMT and co-opt different cadres of health workers as needed.

All counties should invest in forming the target number of CHUs with adequately trained CHVs and CHAs to supervise them. Counties need to invest in the payment of CHVs to reduce attrition. The role of the Community Health Committees needs to be done to improve functionality and sustainability.

### 6.4 Health products and technologies

Counties should ensure that primary care facilities are equipped with basic equipment, diagnostics, and essential medicines as per KEPH standards. Counties should also give autonomy to primary care facilities to order directly from KEMSA or purchase commodities through NHIF reimbursements to reduce drug stockouts. Counties should also address the challenges in commodity supply due to KEMSA's poor fill rate or county debts by engaging with KEMSA or other suppliers or exploring alternative sources of funding. Counties should also invest in training and hiring qualified personnel for commodity quantification and management at primary care facilities.

### 6.5 Monitoring & evaluation

Counties should invest in EMR systems and digital tools to improve health data collection and M&E of PHC programs at national and county levels. Counties should also ensure interoperability of EMR systems with KHIS and scale up EMR systems to all health facilities. Counties should also adopt or adapt PHC-specific indicators as outlined in the National M&E Plan and the PCN guidelines to monitor PCN implementation and performance. Counties should also ensure consistent and high-quality reporting at the community level through the ECHIS system.

## 7 Conclusion

PHC has been shown to be an effective vehicle for achieving UHC. Nonetheless, this requires strong national and subnational leadership, coordination, financial commitment and investment in health system strengthening from the community to the primary level facilities. Different PHC models exist, and one of the most promising models, which can be used to scale up PHC in low- and middle-income countries, is the primary healthcare network (PCN). The model takes advantage of multi-disciplinary teams to provide services from primary-level facilities to the communities. The success of PHC and the achievement of UHC is also nested in sustainable health financing models of which health insurance programs are key. Digital health systems are increasingly becoming important in strengthening PHC by enabling the empanelment of community members to primary link facilities, improved patient referral and continuity of care.
